# Supplementary material for: Nanoscale multistate resistive switching in WO3 through scanning probe induced proton evolution
Source: Nat Commun. 2023 Jul 4;14:3950. doi: 10.1038/s41467-023-39687-9 (PMC10319886; doi:10.1038/s41467-023-39687-9)
Supplement: Supplementary file 1 — Supplementary Info [file 41467_2023_39687_MOESM1_ESM.docx]

**Supplementary information for “Nanoscale multistate resistive switching in WO_3_ through scanning probe induced proton evolution”**

Fan Zhang^1,2^, Yang Zhang^1^, Linglong Li^1^, Xing Mou^3^, Huining Peng^1^, Shengchun Shen^1^, Meng Wang^4^, Kunhong Xiao^1^, Shuai-Hua Ji^1,5^, Di Yi^6^, Tianxiang Nan^3,7^, Jianshi Tang^3,7^ and Pu Yu^1,5^*

^1^State Key Laboratory of Low Dimensional Quantum Physics and Department of Physics, Tsinghua University, Beijing 100084, China.

^2^State Key Laboratory of Information Photonics and Optical Communications & School of Science, Beijing University of Posts and Telecommunications, Beijing 100876, China

^3^School of Integrated Circuits, Beijing National Research Center for Information Science and Technology (BNRist), Tsinghua University, Beijing 100084, China

^4^RIKEN Center for Emergent Matter Science (CEMS), Wako, 351-0198 Japan

^5^Frontier Science Center for Quantum Information, Beijing 100084, China

^6^State Key Laboratory of New Ceramics and Fine Processing, School of Materials Science and Engineering, Tsinghua University, Beijing 100084, China

^7^Beijing Innovation Center for Future Chips (ICFC), Tsinghua University, Beijing 100084, China.

*Email: [yupu@mail.tsinghua.edu.cn](mailto:yupu@mail.tsinghua.edu.cn)

**
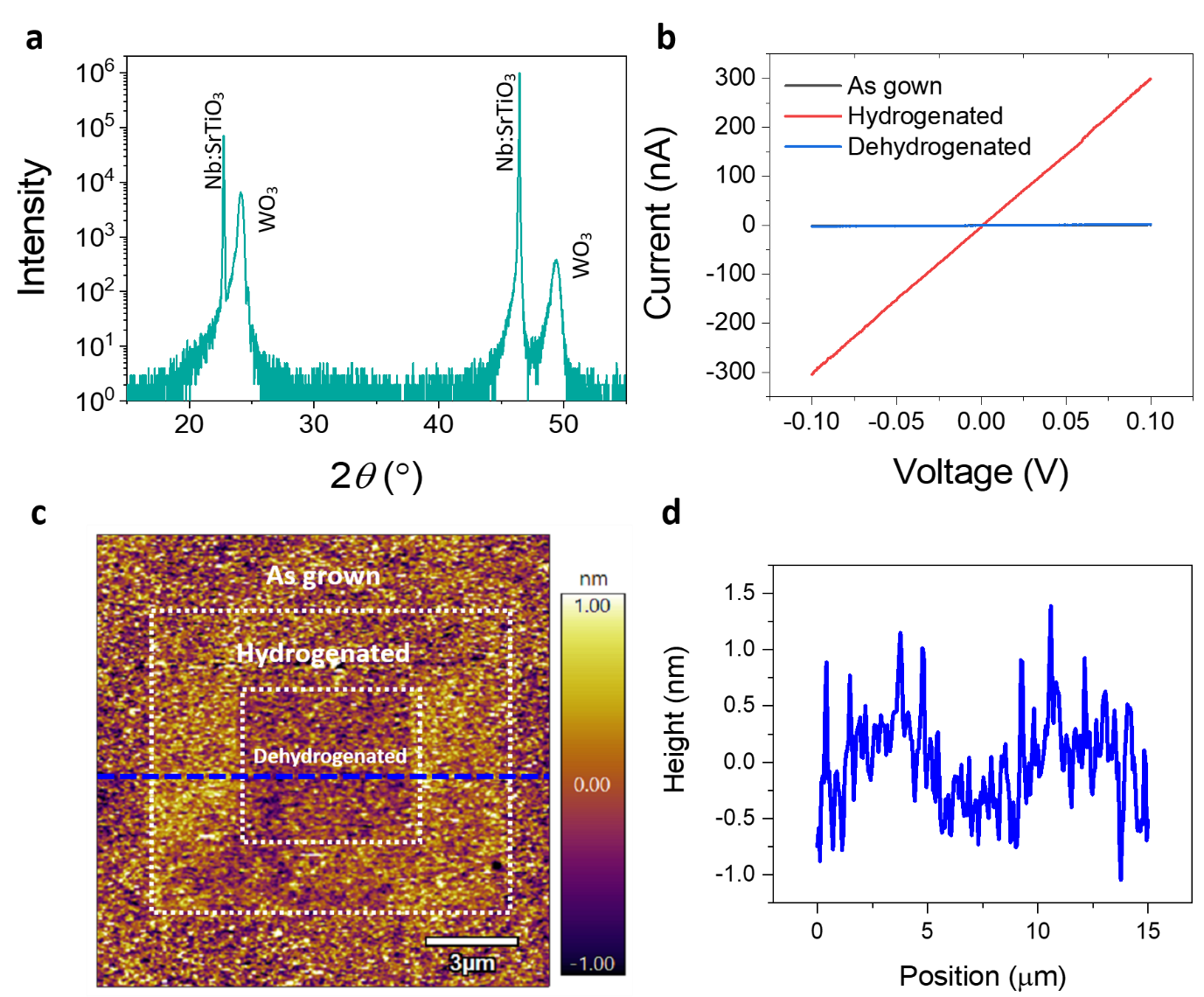
**

**Supplementary Fig. 1 | Structural and electrical characterization of WO_3_ thin films. a,** X-ray diffraction spectra of the as-grown WO_3_ thin film grown on Nb: SrTiO_3_ (001) substrate. **b,** Characteristic local I–V measured with conducting AFM tip. The black, red and blue lines are the I-V curves at as grown, hydrogenated and dehydrogenated regions, respectively. **c,** Topography of a representative WO_3_ thin film through the hydrogenation and dehydrogenation processes. The regions of as-grown, hydrogenated and dehydrogenated are outlined with the dot lines, and the corresponding cAFM image is shown in Fig. 1b. **d**, The topography profile of the line crossing three different areas, as labeled by the blue dash line in (**c**). The hydrogenated area is about 1 nm higher than the non-hydrogenated areas. We note that the slight change in topography should be attributed to the hydrogenation induced lattice expansion. This result is consistent with the results obtained from the TEM analysis, where notable lattice expansion (2.3%) along the c-axis is observed at the hydrogenated region.


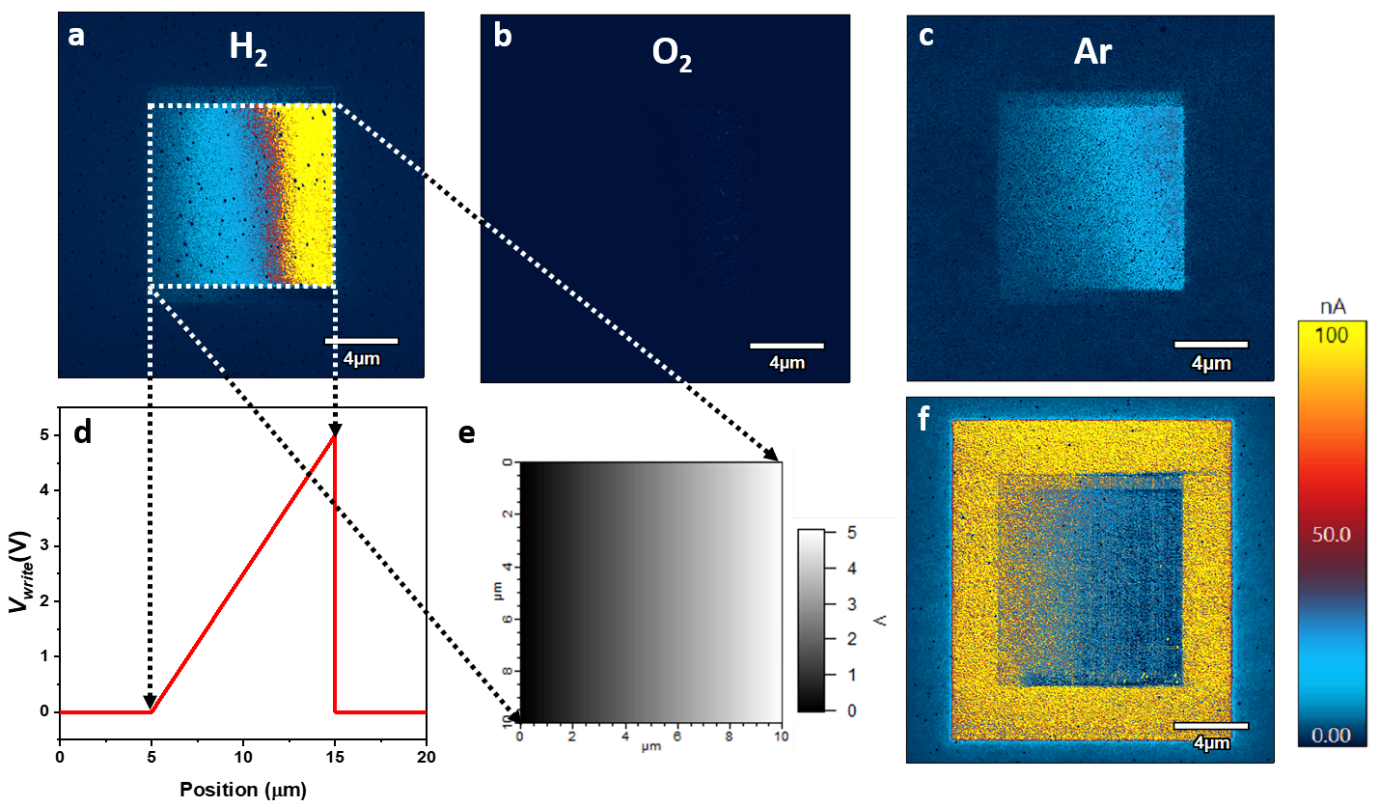


**Supplementary Fig. 2 | Probe induced IMT at various gas environments**. **a-c,** Probe-induced local conductivity modulation carried out in (**a**) forming gas (5% H_2_ + 95% Ar), (**b**) pure O_2_, and (**c**) pure Ar. The writing voltage at the middle region gradually increase from 0 V to 5 V. Data shown here are summarized and plotted in Fig. 1c as current profiles. Compared with the pronounced conductivity observed in (**a**) due to the hydrogenation effect, the measurements at pure O_2_ and pure Ar show much-reduced conductivity. **d**, Biased voltage vs. position used for the writing process of conducting maps shown in (**a-c**). **e**, Greyscale voltage template used for the lithographic hydrogenation in (**a-c**). **f**, Voltage-dependent dehydrogenation process from a hydrogenated sample. The application of negative biased voltages dehydrogenates the sample with the recovery of an insulating state. All conducting maps shown here employ the same color scale bar as shown on the right.


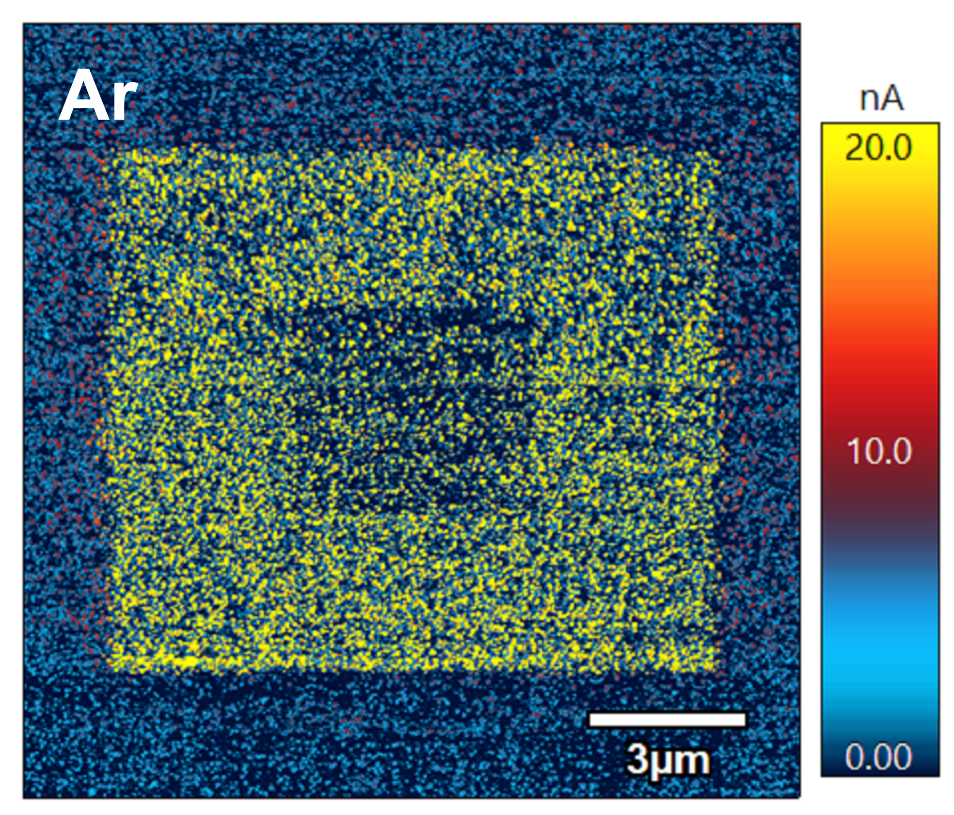


**Supplementary Fig. 3 | Reverse switching test of metallic state obtained through oxygen vacancy formation.** The result reveals that the oxygen vacancy induced metallic state cannot be fully recovered, which is due to the fact that the oxygen source within the argon environment is insufficient to compensate for the oxygen vacancies.


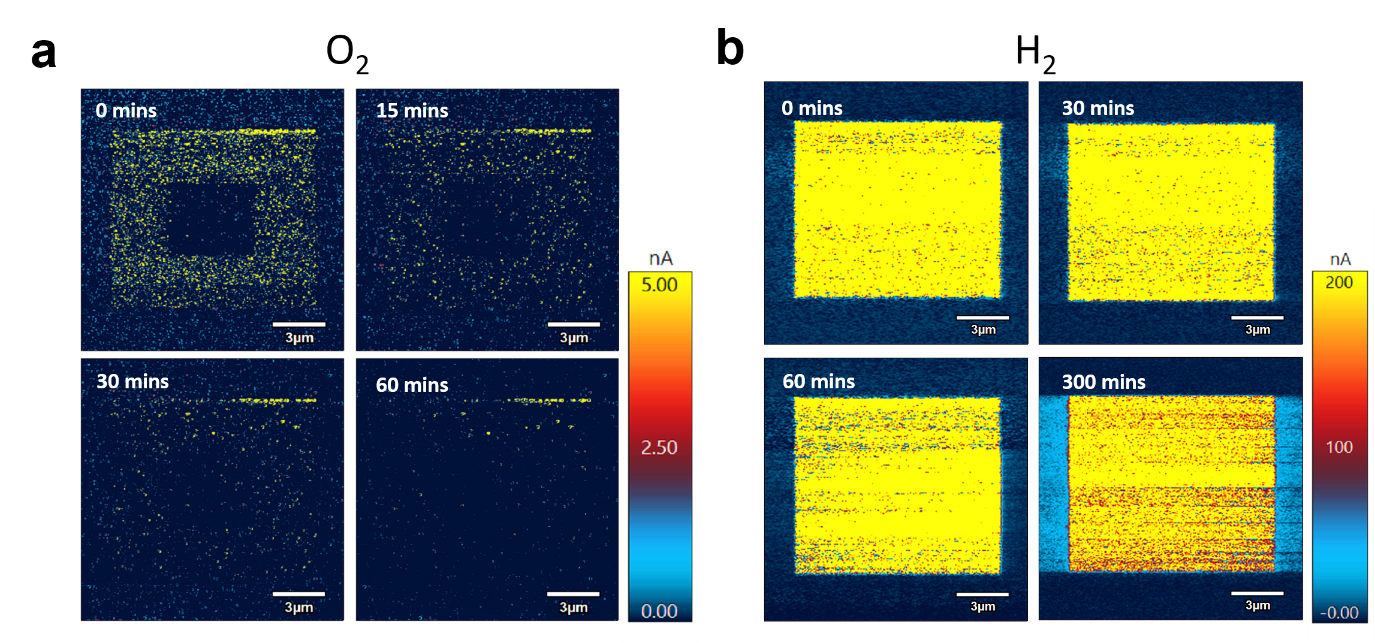


**Supplementary Fig. 4 | Reversible IMT and** **retention tests at various gas environments.** **a**, Retention test in O_2_. Compared with Ar gas, the erasing effect (central area) becomes more obvious within the oxygen gas environment (figure of 0 mins). However, the conducting pattern disappears rapidly within 1 hour; after that, the whole sample returns to an insulating state. This result suggests that although the oxygen-rich environment can stimulate more efficient oxygen exchange, the oxygen vacancies within the sample are compensated spontaneously with the oxygen gas, leading to poor retention. **b**, Retention test in forming gas (5% H_2_ + 95% Ar). It shows that the hydrogenated area sustains a high conductivity state up to 300 minutes after the hydrogen intercalated.


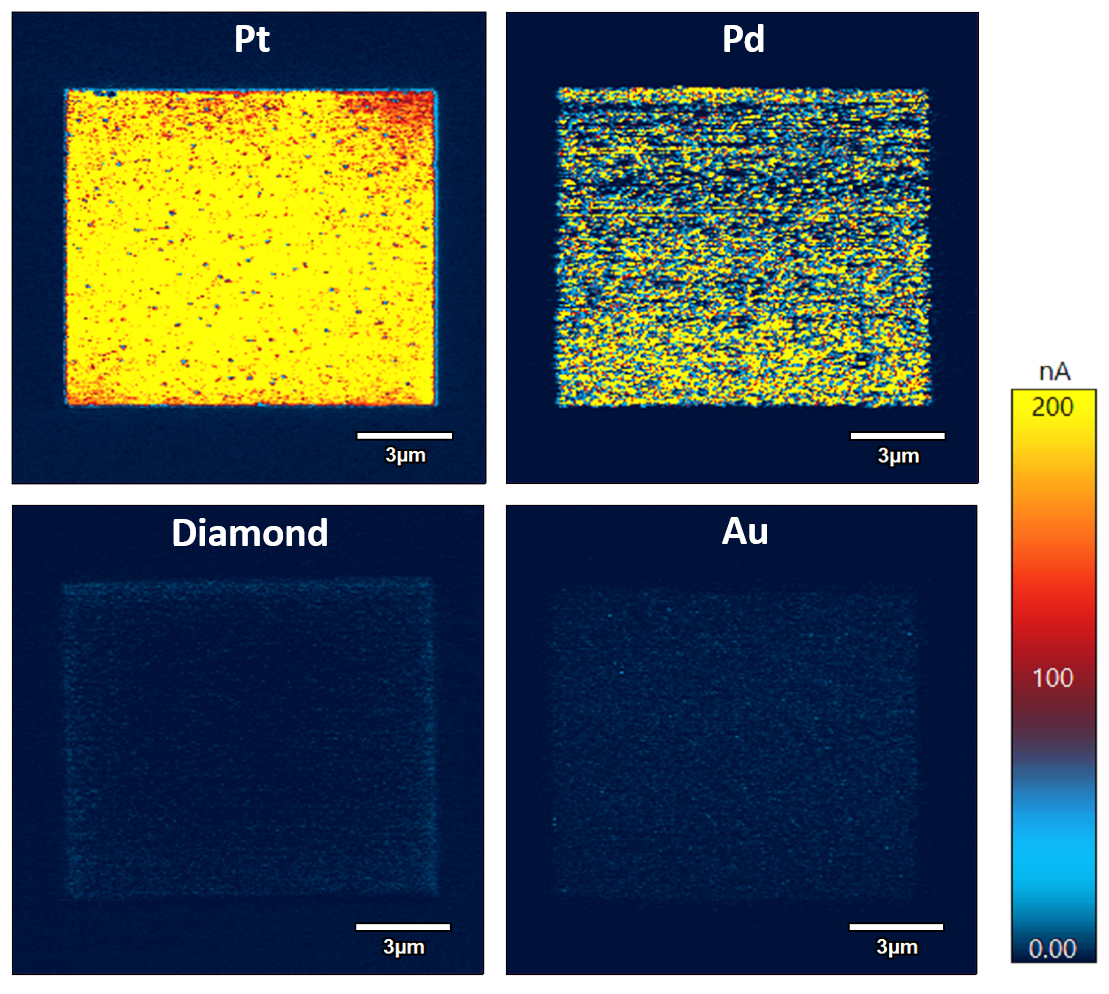


**Supplementary Fig. 5 | Probe-dependent tests for the biased-probe-induced IMT.** An identical biased voltage of +4V was employed for all these measurements, and the gas environment was forming gas. The diamond (DCP 200, NT-MDT) and Pt-coated probes (HQ:NSC18/Pt, MikroMasch) are commercially available, while the Pd- and Au- coated probes were prepared by coating the corresponding metals on nonconductive silicon-made AFM probes (HQ:NSC18/Cr-Au BS, MikroMasch) through sputtering. The comparison reveals that the resistance changes at the regions patterned with diamond and Au-coated probes are much smaller than those with the Pt- and Pd- coated probes. This result suggests that the catalytic metals (i.e., Pt, Pd) play essential roles in the hydrogenation process to achieve the IMT.


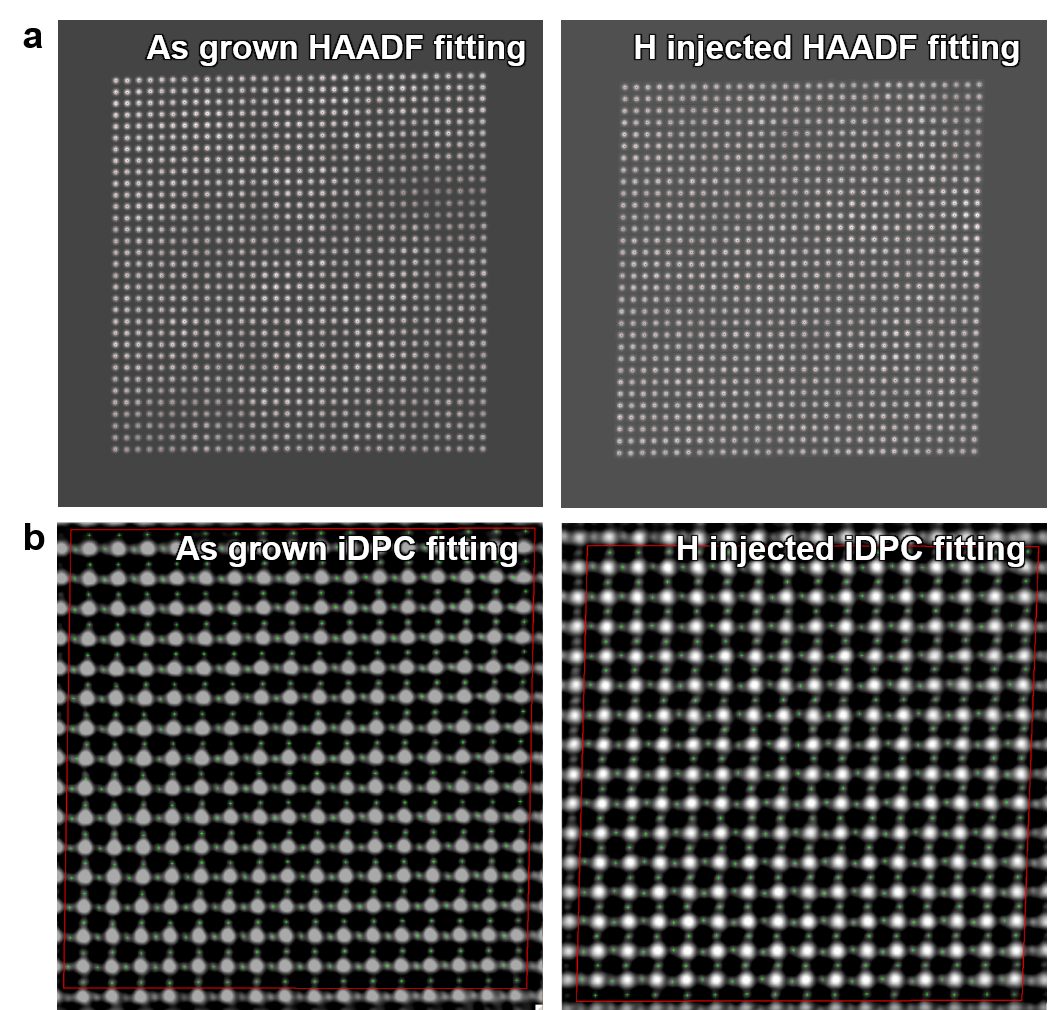


**Supplementary Fig. 6 | Crystalline structural analysis for the as-grown and H-injected (hydrogenated) WO_3_ samples. a,** Fitting results of the HAADAF images to obtain the lattice constants shown in Fig. 2c. **b,** Fitting results of the iDPC images used for the measurement of oxygen concentration shown in Fig. 2f. Red and green points in (**a**) and (**b**) represent the atomic positions of W and O atoms determined through Gaussian fitting.


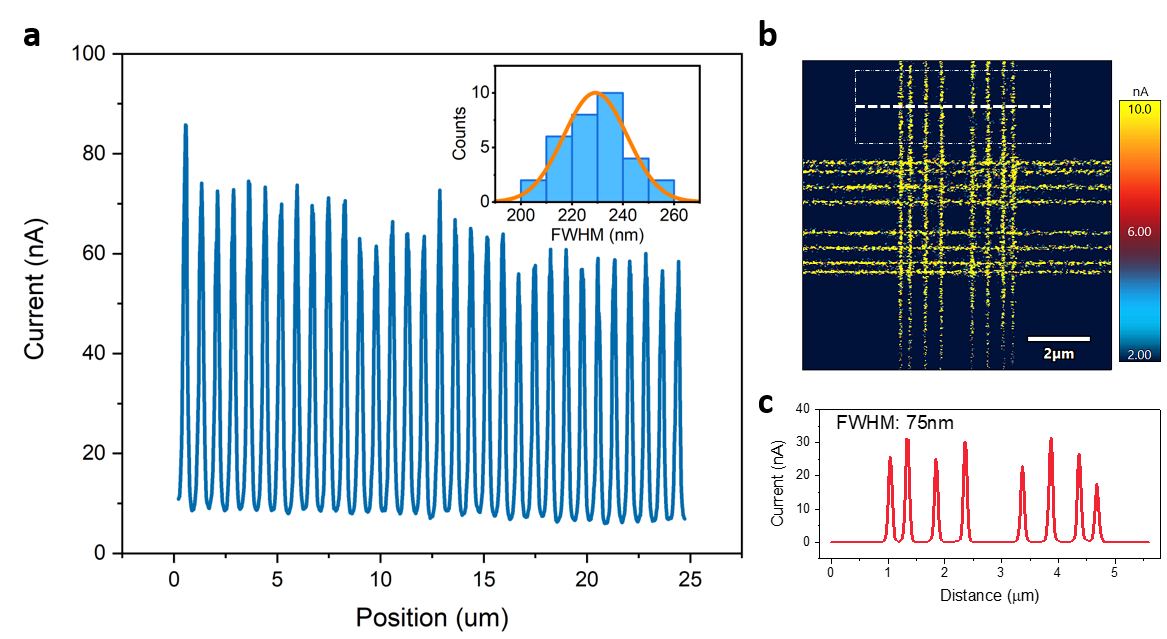


**Supplementary Fig. 7 | Extended data for the conductive wires.** Current profile for the patterned conductive wires shown in **Fig. 3c**. The inset is the statistical FWHM of the 32 illustrated peaks. **b**, Patterned matrix formed with conducting wires written with 2 V.  **c**, Profile across the conducting wires marked with dash line in **b**. The average FWHM for these wires is estimated to be about 75 nm, which is the characteristic length scale for the tip-induced hydrogenation within WO_3_ thin films.


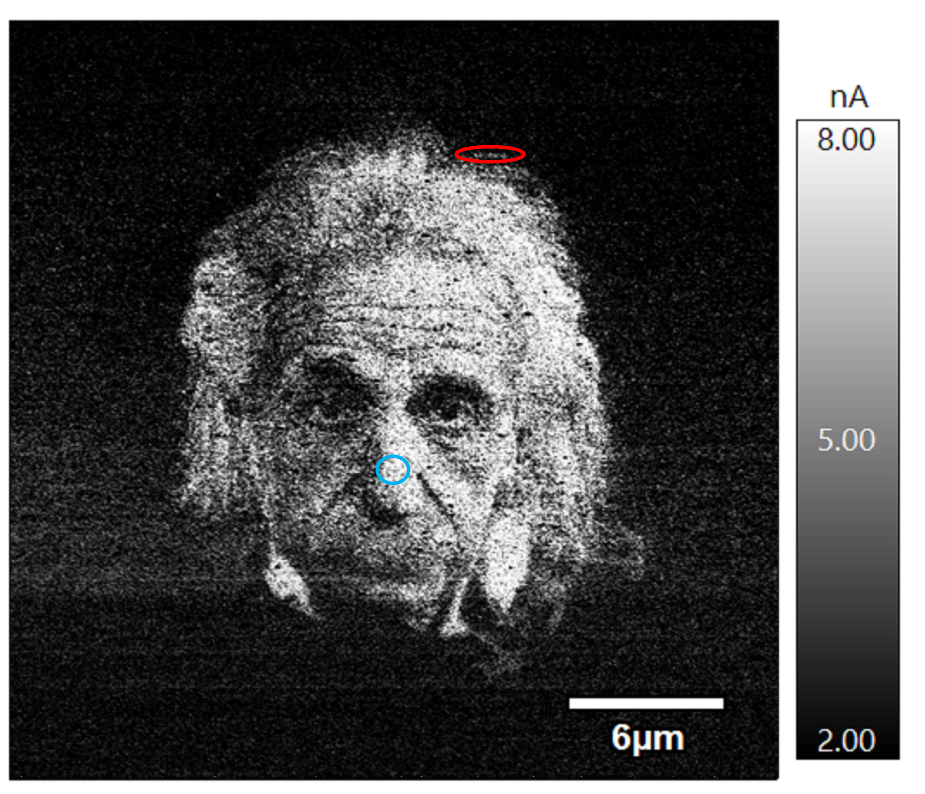


**Supplementary Fig. 8 | The portrait of Albert Einstein through probe-induced hydrogenation.** The writing voltages at Einstein’s hair in the red circle and nose in the blue circle are about 0.87 V and 2.73 V, respectively.


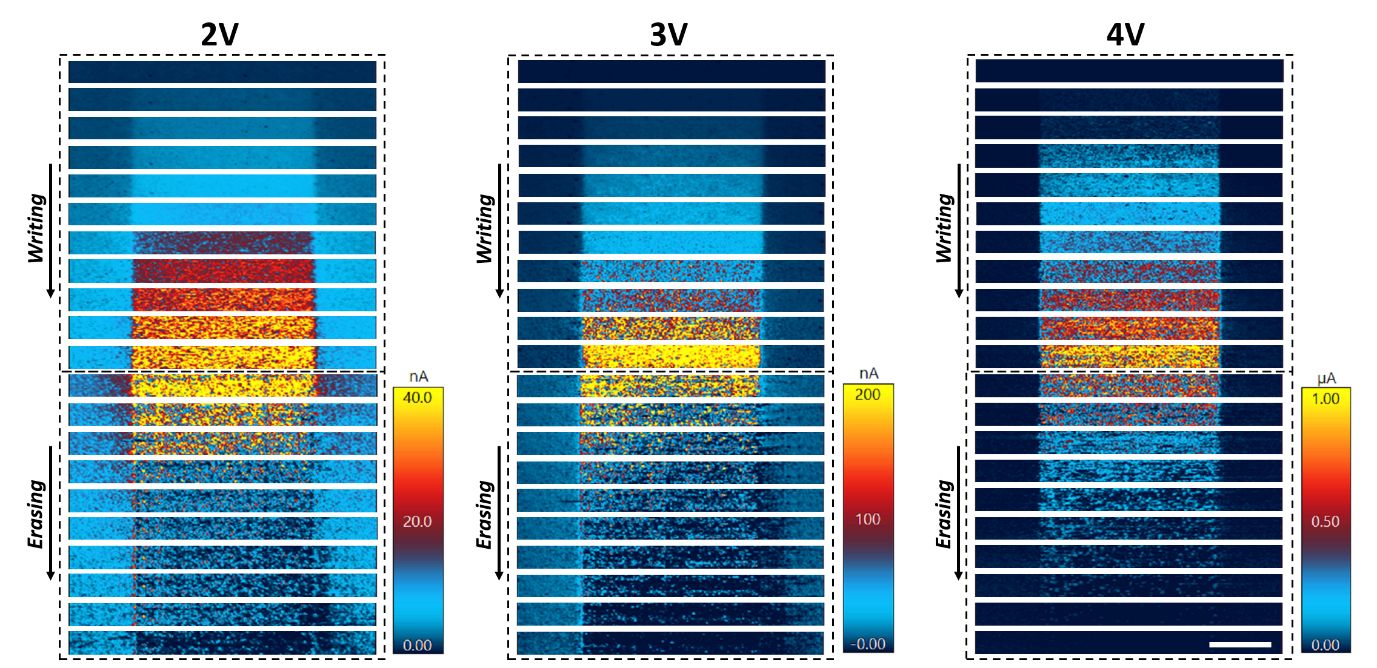


**Supplementary Fig. 9 | Evolution of local conductance at identical regions through successive writing (hydrogenation) and erasing (dehydrogenation) processes.** Three representative voltages (i.e., 2 V, 3 V and 4 V) were employed during the measurements. The scan rate is fixed at 9.8 Hz. The writing and erasing voltages have the same magnitude but opposite polarity. Identical regions were written successively 10 times in each process and then erased 10 times with the noted voltages. The data for 2 V and 3 V are summarized in **Fig. 4b** of the main text. The scale bar illustrates the length of 2 μm.


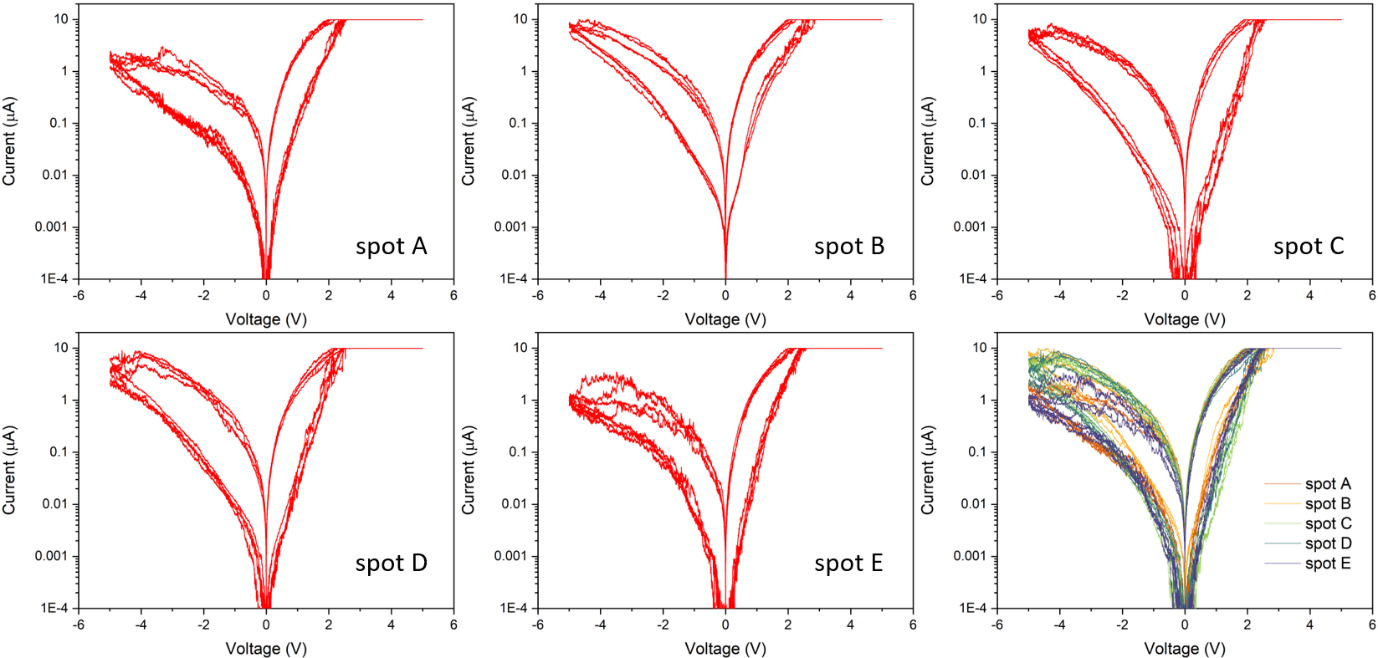


**Supplementary Fig. 10 | I-V measurement at 5 randomly chosen spots on the same sample.** The last figure is an overlay of these loops.
